# Microencapsulation of Spent Coffee Extract Within *Saccharomyces cerevisiae* Cells via Spray Drying and Evaluation of Its In Vitro Bioaccessibility

**DOI:** 10.3390/foods14061053

**Published:** 2025-03-19

**Authors:** Isabel H. Chacón-Figueroa, Ramón F. Dórame-Miranda, Guadalupe A. López-Ahumada, Carmen L. Del-Toro-Sánchez, Maribel Ovando-Martínez, Nohemí Gámez-Meza, Fernando Martínez-Bustos, José C. Rodríguez-Figueroa, Jesús Enrique Gerardo-Rodríguez, Kristin Whitney, Ariadna Thalía Bernal-Mercado, Maribel Plascencia-Jatomea, Víctor M. Herrera-Jiménez

**Affiliations:** 1 Department of Food Research and Graduate Program (DIPA), University of Sonora, Hermosillo C.P. 83000, Sonora, Mexico; a216214920@unison.mx (I.H.C.-F.); amanda.lopez@unison.mx (G.A.L.-A.); carmen.deltoro@unison.mx (C.L.D.-T.-S.); jesusenrique.gerardo@unison.mx (J.E.G.-R.); thalia.bernal@unison.mx (A.T.B.-M.); maribel.plascencia@unison.mx (M.P.-J.); 2 Department of Scientific and Technological Research (DICTUS), University of Sonora, Hermosillo C.P. 83000, Sonora, Mexico; maribel.ovando@unison.mx (M.O.-M.); nohemi.gamez@unison.mx (N.G.-M.); 3 Centro de Investigación y de Estudios Avanzados, Instituto Politécnico Nacional, Libramiento Norponiente, Fracc. Real de Juriquilla, Querétaro C.P. 76230, Querétaro, Mexico; fmartinez@cinvestav.mx; 4 Department of Chemical Engineering and Metallurgy, University of Sonora, Hermosillo C.P. 83000, Sonora, Mexico; jose.rodriguez@unison.mx; 5 Whistler Center for Carbohydrate Research, Department of Food Science, Purdue University, West Lafayette, IN 47907, USA; klwhitne@purdue.edu; 6 Department of Industrial Engineering, University of Sonora, Hermosillo C.P. 83000, Sonora, Mexico; victor.herrera@unison.mx

**Keywords:** antioxidants, bioactive compounds, coffee, plasmolyzed yeast cells, functional beverage, chlorogenic acid

## Abstract

Spent coffee is rich in bioactive compounds, including chlorogenic acid, caffeic acid, and caffeine, which offer health benefits. However, digestive processes can degrade these compounds; therefore, microencapsulation within *Saccharomyces cerevisiae* yeast cells offers a novel method to stabilize these bioactive compounds during digestion. In fact, it is important to mention that this technique of microencapsulation in *Saccharomyces cerevisiae* has not been previously applied to spent coffee extract. As a result, in this study, spent coffee extract was microencapsulated in non-plasmolyzed (NPCs) and plasmolyzed (PCs) yeast cells using the spray drying method. The physicochemical properties of the extract and the microencapsulates were characterized, and the bioaccessibility of the bioactive compounds was evaluated with digestion in vitro. Encapsulation efficiency (EE) was 38.62% for NPCs and 55.78% for PCs, with loading capacities (LCs) of 126.36 and 242 g/kg, respectively (according to Equations (1) and (2)). The presence of antioxidant compounds, identified by HPLC in spent coffee, was confirmed in the microencapsulates using FTIR. In vitro digestion assays revealed higher bioaccessibility of bioactive compounds in the intestinal phase, greater than 90%, and increased antioxidant activity in beer made with plasmolyzed microcapsules (BPM). These results suggest that yeast microencapsulation effectively stabilizes the bioactive compounds of spent coffee extract, releasing them throughout the gastrointestinal tract in vitro, mainly in the intestinal phase. Thus, microencapsulated compounds could serve as functional additives with a good percentage of intestinal bioaccessibility.

## 1. Introduction

Coffee is one of the world’s most consumed beverages and is second in popularity after black tea [1]. Spent coffee is the waste obtained from the extraction process of soluble coffee and the infusion drink of the ground beans. This waste represents more than 50% of the total weight of the roasted coffee bean, making it one of the most important wastes, as more than 6 million tons per year are generated worldwide [2,3]. The large volumes of spent coffee generated can damage the environment. Therefore, there is growing interest in using spent coffee, particularly because it contains bioactive compounds such as caffeic acid, chlorogenic acid, and caffeine. The bioactive compounds in spent coffee have antioxidant activity, and moderate consumption helps prevent chronic non-communicable diseases [4]. However, the bioactive compounds of spent coffee are susceptible to deterioration due to environmental and chemical factors, such as temperature, pH, humidity, and metal ions. Therefore, to take greater advantage of the bioactivity of these compounds, microencapsulation techniques can be applied to stabilize them by selecting an appropriate and compatible encapsulation material [5,6]. Yeast cells as a delivery system for bioactive compounds have already been studied due to the wide range of advantages they offer, such as the high-volume and low-cost microencapsulation process and the safety of yeast cells for human nutrition (GRAS material), in addition to being commercially available [7,8]. Among the yeast species, one of the most prominent is *Saccharomyces cerevisiae* due to its cell wall being made up of a cross-linked network of mannoproteins and fibrous β-1,3-glucans, which helps protect the encapsulated compounds against harmful factors [8,9]. Microencapsulation is a technique that has been applied in the food industry; various studies have demonstrated the efficiency of *Saccharomyces cerevisiae* yeast cells in encapsulating essential oils, enzymes, terpenes, alkaloids, and phenolic compounds [10,11,12,13,14]. The encapsulation efficiency of mostly hydrophilic compounds such as phenolic acids has increased with pretreatments such as plasmolysis, which changes the lipid composition of the plasma membrane, increasing permeability without significant alterations in the cell wall, improving intracellular spaces and decreasing viability. This allows for its application in fermented products such as yogurt. Rubio et al. [15] added microcapsules of *S. cerevisiae* loaded with grape pomace colorant to yogurt, demonstrating the use of enriched yeasts as interesting colorants and additives for the production of fermented foods, thus opening up the possibility of their application in beer.

Beer is a complex and ancient alcoholic beverage and is the most fermented beverage consumed worldwide. Around 800 million hectoliters of beer are manufactured annually, increasing by 14.3% in 2023 [16]. Beer is composed of carbohydrates, minerals, vitamins, amino acids, ethyl alcohol, and phenolic compounds. The phenolic compounds in beer come from malt and hops (in a proportion of 70 and 30%, respectively), which provide the sensory and biological properties of the drink [17]. However, these compounds decrease during the brewing process due to the high temperature, lautering, and filtering. While ethanol can increase the concentration and profile of phenolic compounds in beer, moderate ethanol consumption is a maximum of 12 g of ethanol/day for women and 24 g of ethanol/day for men [18,19]. There is currently a greater demand for the development of beers that, in addition to meeting nutritional needs, also have added value to the consumer’s health; hence, the incorporation of functional additives has gained popularity worldwide [20]. The addition of microcapsules in beer could increase antioxidant activity and bioabsorption in the gastrointestinal tract, increasing the benefits of bioactive compounds [21,22]. This work aimed, for the first time, to microencapsulate spent coffee extract by means of plasmolyzed and non-plasmolyzed *Saccharomyces cerevisiae* yeast cells. Accordingly, the study evaluated the protective effect of these yeast cells when used as encapsulating materials and investigated their potential novel applications as functional additives in craft beer. Moreover, the bioaccessibility of the bioactive components was evaluated both during and after an in vitro digestion process.

## 2. Materials and Methods

### 2.1. Materials

The spent coffee was obtained from a Starbucks^®^ commercial coffee shop in Hermosillo, Sonora (2022). The stout-style craft beer was purchased from a craft brewery, “La Hache”, in Hermosillo, Sonora (2024). The *Saccharomyces cerevisiae* yeast cells were from the S-04 strain of the Fermentis^®^ brand. The solvents used for chromatography (HPLC) analysis were methanol, formic acid, and ethanol (JT Baker, Xalostoc, Mexico City, Mexico). The standards are from Sigma Aldrich Co. (St. Louis, MO, USA), and the Milli-Q water is from EMD Millipore Corporation (Billerica, MA, USA). All reagents were of analytical grade.

### 2.2. Extraction of Bioactive Compounds from Spent Coffee

As previously reported, the extraction of bioactive compounds from spent coffee was obtained through optimized conditions with the sonication method using an ultrasonic bath, according to [23].

### 2.3. Identification and Quantification of Antioxidants in Spent Coffee by HPLC

The identification of bioactive compounds from spent coffee extract was carried out with some variations [24] using high-performance liquid chromatography equipment and a diode array detector (HPLC-DAD, 1260 Infinity model, Agilent Technologies, Inc., Santa Clara, CA, USA). The dried spent coffee extract was resuspended in 30% (*v*/*v*) ethanol HPLC-grade for the injection. The samples were filtered with a nylon filter (0.22 μm) and subsequently injected into the system (50 μL). Separation of the molecules was performed using a reverse phase with a C-18 HPLC column (5 μm, 25 cm × 4 mm, Supelcosil^TM^ LC-18, SUPELCO) (Supelco Inc., Bellefonte, PA, USA), and a solvent flow of 0.500 mL/min was used. Mobile phase A was water acidified with 5% formic acid, while mobile phase B was methanol. Elution was performed using a linear gradient from 100 to 98% initial A maintained for 0–2 min, up to 68% A for 30 min, from 68 to 60% A for 0–8 min, from 60 to 5% A for 10 min, and 5% A for 5 min. Bioactive compounds were identified and quantified using calibration curves of secondary standards (gallic acid, sinapinic acid, ferulic acid, caffeic acid, syringic acid, p-coumaric acid, chlorogenic acid, and caffeine). The method was validated by the limit of detection (LOD) and the limit of quantification (LOQ) using linear regression with a concentration starting at 10 mg/mL of each standard analyzed.

### 2.4. Treatment of Saccharomyces cerevisiae Yeast Cells

Yeast cellular activation was performed using a YM broth medium. It was incubated at 28 °C for 72 h. The yeasts were isolated using YM broth with agar. The morphological characteristics of the yeasts were examined using an optical microscope and a methylene blue stain. The resulting biomass was concentrated by centrifugation at 5000× *g* for 15 min at 4 °C (Heraeus Megafuge 16R Centrifuga, Thermo Scientific, Mexico City, Mexico) and stored refrigerated at 4 °C.

### 2.5. Non-Plasmolyzed Saccharomyces cerevisiae Yeast Cells

Non-plasmolyzed cells were treated according to [25] with variations. Yeast cells were placed with sodium phosphate buffer solution (pH 6.8) and washed 5 times by centrifugation with sterile Milli-Q water at 5000× *g* for 15 min at 4 °C (Heraeus Megafuge 16R Centrifuge, Thermo Scientific, Mexico City, Mexico). The samples were stored at −20 °C for subsequent freeze-drying (FreeZone 4.5, Labconco, Kansas City, MO, USA).

### 2.6. Plasmolysis of Saccharomyces cerevisiae Yeast Cells

The process of plasmolyzed cells was carried out according to [25]. The cells were suspended in a hypertonic 10% (*w*/*v*) sodium chloride (NaCl) solution and shaken on a plate (SuperNova model SP135935Q, Thermo Scientific^TM^, Waltham, MA, USA) at 180 rpm for 48 h at 55 °C. The cells were washed 5 times by centrifugation (5000× *g* for 15 min at 4 °C) and frozen at −20 °C to be dried by lyophilization (FreeZone Lyophilizer model 7750020, Labconco, Kansas City, MO, USA).

### 2.7. Microencapsulation of Spent Coffee Extract in Saccharomyces cerevisiae Yeast

Microencapsulation of bioactive compounds was carried out according to [25] with modifications. A ratio of 1:2 (extract:yeast) was added to water to homogenize on a magnetic plate (SuperNova model SP135935Q, Thermo Scientific^TM^, Waltham, MA, USA) at 180 rpm and 40 °C for 12 h (the extract:yeast relationship was made because if there was a greater concentration of yeast, it could be guaranteed that the entire extract is encapsulated). Finally, the solution was dried with a spray dryer (LabPlant SD-Basic spray dryer, LabPlant, Huddersfield, UK) with an initial or inlet temperature of 105 °C and a final or outlet temperature of 55 °C. The feed flow was 6 mL/min, the compressed air pressure was 35 psi, and the nozzle assembly was 0.5 mm with a rotatory atomization system. The dried microcapsules obtained were stored at −20 °C.

### 2.8. Scanning Electron Microscopy (SEM)

The morphological characteristics and diameter were studied using a scanning electron microscope (FESEM model JEOL, JSM-7610F, Tokyo, Japan) operated at 15 kV. The samples were coated with a gold (Au)/palladium (Pd) mixture in an argon gas vacuum chamber.

### 2.9. Fourier Transform Infrared Spectroscopy (ATR-FTIR)

The spectra of the spent coffee and microencapsulated extracts were obtained using an ATR-FTIR spectrometer (Spectrum GX, Perkin Elmer, Waltham, MA, USA) in a range of 4000 to 400 cm^−1^ wavenumber and 4 cm^−1^ resolution with 32 scans.

### 2.10. Differential Scanning Calorimetry (DSC)

A total of 10 mg of sample was placed in aluminum capsules in the calorimetry equipment (DSC822e, refrigerated, Mettler Toledo Lab Plant, Huddersfield, UK) and subsequently subjected to a temperature scan from 25 to 350 °C under a nitrogen atmosphere at a flow rate of 25 mL/min and a heating rate of 5 °C/min.

### 2.11. Encapsulation Efficiency (EE) and Load Capacity (LC)

The evaluation of the loading capacity and encapsulation efficiency of the spent coffee extract in the microencapsulates was carried out by the method detailed in [26] with some variations. The cells were washed with 30% ethanol and subsequently pelleted with a centrifuge at 5000× *g* at 4 °C for 15 min (Heraeus Megafuge 16R Centrifuga, Thermo Scientific, Mexico City, Mexico). The supernatant was then used to determine the concentration of total surface phenols (mg EAG/g). On the other hand, the cells were sonicated in an ultrasonic bath (Branson Sonicator, model 1510, Danbury, CT, USA) with 30% ethanol for 10 min at 30 °C and centrifuged at 5000× *g* for 15 min at 4 °C (Heraeus Megafuge 16R Centrifuge, Thermo Scientific, Mexico City, Mexico). The total phenol inside the microcapsule was quantified (mg EAG/g). The encapsulation efficiency was determined with Equation (1) and the loading capacity with Equation (2) [27].

Encapsulation efficiency (%) = (Microencapsulated total phenols)/(Surface phenols) × 100 (1)

Load capacity = (Total encapsulated phenols − surface phenols)/(Total extract phenols) × 100(2)

### 2.12. Gastrointestinal Digestion In Vitro

For the digestion test of antioxidant compounds, it was necessary to simulate the conditions of gastric and intestinal digestion using the static in vitro simulation method of gastrointestinal food digestion by the INFOGEST 2.0 standardized process [28]. The samples used were non-additive beer (NAB), beer with spent coffee extract (BSCE), beer with non-plasmolyzed microcapsules (BNPM), and beer with plasmolyzed microcapsules (BPM). The coffee extracts were 100 mg of spent coffee extract powder mixed with 10 mL of beer and 200 mg of microcapsule powder with 10 mL of beer, according to a previous study, as was the concentration level of phenols in the extract and the microencapsulates [23].

#### 2.12.1. Gastric Phase (GP)

A total of 8 mL of the GP saline solution with 5 mL of sample was adjusted to pH 3, and 0.5 mL of porcine pepsin (300 mg/mL) (2000 U/mL, Sigma Aldrich, CAS 9001-75-6) and 0.5 mL of porcine pancreas lipase (7 mg/mL) (60 U/mL, Sigma Aldrich, CAS 9001-62-1) were added. It was incubated for 2 h at 37 °C (Thermo Scientific 2864 Precision Circulating Water Bath 51221035, Marietta, OH, USA).

#### 2.12.2. Intestinal Phase (IP)

A total of 8.5 mL of IP salt solution was added to the tubes resulting from the GP, and the pH was readjusted to 7. A total of 2.5 mL of bile salts (7 mg/mL) (10 mM, Sigma Aldrich, CAS 8008-63-7) and 5 mL of porcine pancreatin (5 mg/mL) (trypsin activity 100 U/mL) were also added. It was incubated for 2 h at 37 °C (Thermo Scientific 2864 Precision Circulating Water Bath 51221035, Marietta, OH, USA).

#### 2.12.3. Dialysis (D)

The absorption of phenolic compounds by passive diffusion was simulated. The digest of the intestinal phase was placed on cellulose membranes. Quantification was carried out to obtain the percentage of bioaccessibility of the phenolic compounds in the digestive phases using Equation (3). The quantification of the total phenols from dialysis was carried out with Equation (4).

Bioaccessibility (%) = (Digestive phase phenols)/(Undigested phenols) × 100(3)

Bioaccessibility (%) = (Undigested phenols − dialysis phenols)/(Undigested phenols) × 100(4)

### 2.13. Liquid–Liquid Extraction of Bioactive Compounds After the Digested

An extraction of the bioactive compounds from each digest [29] was carried out: 2 mL of each digest was placed with 2 mL of ethyl acetate:ethyl ether (50:50) and homogenized with vortex for 30 s. Subsequently, the organic phase was recovered, and the solvent was removed with nitrogen gas (N_2_). It was resuspended with 30% (*v*/*v*) ethanol for subsequent analyses.

### 2.14. Total Phenols

The analysis of total phenols was carried out using the methodology proposed by [30] with modifications. A total of 30 µL of the sample, 150 µL of Folin-Ciocalteu reagent 0.1 N, and 120 µL of sodium carbonate (Na_2_CO_3_) at 7.5% (*w*/*v*) were placed in the microwell. Absorbance was read at 750 nm (Veloskan™ LUX Multimode Microplate Reader, Thermo Scientific, Waltham, MA, USA) after 30 min in darkness. The results were reported as mg GAE per g for dry samples and mg GAE/L for liquid samples. The range of the standard curve is 0 to 0.4 mg/mL, R^2^ > 0.99.

### 2.15. Trolox Equivalence Antioxidant Capacity Assay

The antioxidant capacity of Trolox equivalents was determined following [31] with some changes. A total of 280 µL of ABTS radical was placed with 20 µL of sample in microwells. The absorbance was analyzed in a microplate reader at 734 nm (Veloskan™ LUX Multimode Microplate Reader, Thermo Scientific, Waltham, MA, USA) after 7 min in the dark. The antioxidant capacity was reported in µMol Trolox equivalents (TE)/g dry weight and as mMol TE/g or L. The range of the standard curve is 0 to 0.5 mg/mL, R^2^ > 0.99.

### 2.16. DPPH Radical Scavenging Capacity Test

The DPPH test was carried out according to [32] with some modifications. The DPPH radical (0.008 mg/mL) was prepared in reagent-grade methanol, and the initial absorbance was adjusted to 0.7 ± 0.02. 20 µL of the sample. A total of 280 µL of DPPH radical was placed in a microplate, and the absorbance was obtained at 515 nm after 30 min in darkness in a microplate reader (Veloskan™ LUX Multimode Microplate Reader, Thermo Scientific, Waltham, MA, USA). The results were reported as µMol TE/g and mMol TE/g or L. The range of the standard curve is 0 to 0.4 mg/mL, R^2^ > 0.99.

### 2.17. FRAP Test

The FRAP test was carried out according to [33] with some variations. A total of 20 μL of the sample was placed in a microplate and mixed with 280 μL of the FRAP mixture. The absorbance was measured at 630 nm after 30 min in darkness. The results were reported as µMol Trolox equivalents (TE)/g dry weight and mMol TE/g or L. The range of the standard curve is 0 to 0.4 mg/mL, R^2^ > 0.99.

### 2.18. Statistical Analysis

A completely randomized design was carried out, and all treatments were carried out in triplicate using an analysis of variance (ANOVA). A comparison of means was performed using the Tukey test (*p* ≤ 0.05) and a Pearson correlation of means. InfoStat statistical software Version 2020 was used. The graphs were made in the SigmaPlot 12.0 program.

## 3. Results

### 3.1. Identification and Quantification of Bioactive Compounds

Table 1 shows the compounds that were identified. The main compound was caffeine (28,919.13 ± 4.18 µg/g), followed by chlorogenic acid (4349.42 ± 4.66 µg/g) and caffeic acid (880.14 ± 4.07 µg/g). The compounds that were identified in a lower concentration were simple phenolic compounds such as gallic acid (448.02 ± 5.74 µg/g) and syringic acid (412.87 ± 2.89 µg/g). In addition, hydroxycinnamic acids were also identified in low concentrations, such as p-coumaric acid (583.91 ± 6.34 µg/g), ferulic acid (484.23 ± 6.83 µg/g), and sinapinic acid (787.19 ± 4.66 µg/g). The presence of these antioxidants in the spent coffee extract is due to the biosynthesis of secondary metabolites during the growth of the coffee plant under stress. Furthermore, the concentration of the compounds present is attributed to the type of drink made before the generation of the by-product since the more compounds left in the drink, the lower their concentration will be in the residue [34,35]. The presence of phenolic compounds and caffeine in spent coffee has already been reported, such as in [36], which obtained a caffeine concentration of 54,440.27 µg/g, 613.61 µg/g of chlorogenic acid, and 204.95 µg/g of caffeic acid. Compared to the present investigation, the authors reported a higher concentration of caffeine but a lower concentration of chlorogenic acid and caffeic acid. On the other hand, ref. [37] reported a caffeine concentration of 3929 µg/g, which is lower than that reported in this investigation. The differences are due to possible changes in the cultivation method, obtaining the by-product, and the extraction process of the bioactive compounds [38]. The results support spent coffee as a promising raw material for extracting bioactive compounds such as hydroxycinnamic acids, chlorogenic acid, and caffeine. Chlorogenic acid is a potent antioxidant that shows effects for mitigating unbalanced intracellular redox states. In vitro assays have also reported an anti-inflammatory effect due to a decrease in reactive nitrogen species (RNS), reactive oxygen species (ROS), and the NF-κB signaling pathway [39]. Previous studies have reported that chlorogenic acid was bioaccessible across the intestinal barrier but to a low degree [40]. Therefore, it is important to look for technologies that help increase absorption.

### 3.2. Scanning Electron Microscopy of Yeast Cells and Microcapsules

**Figure 1:**
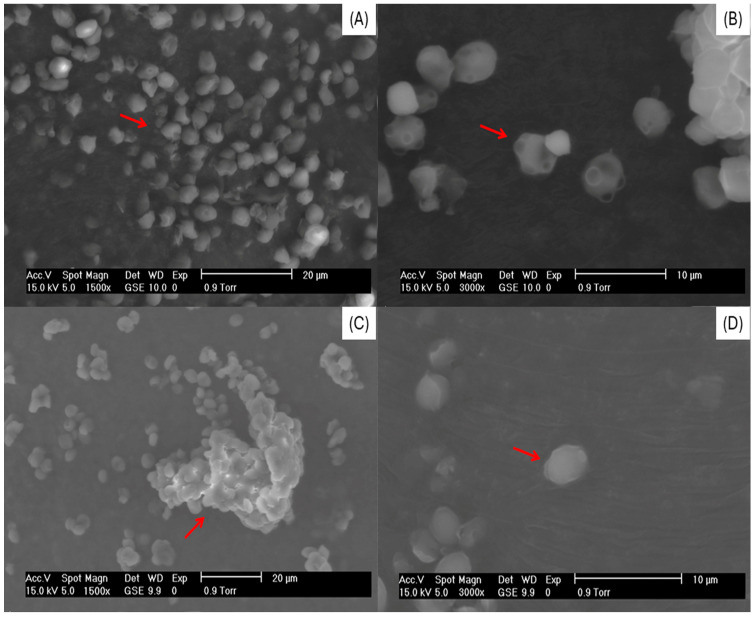
Non-plasmolyzed *Saccharomyces cerevisiae* yeast cells at 1500× (**A**) and 3000× (**B**) and *Saccharomyces cerevisiae* yeast cells plasmolyzed at 1500× (**C**) and 3000× (**D**). The red arrows in the figure indicate the presence of changes in the yeast cells.

**Figure 2:**
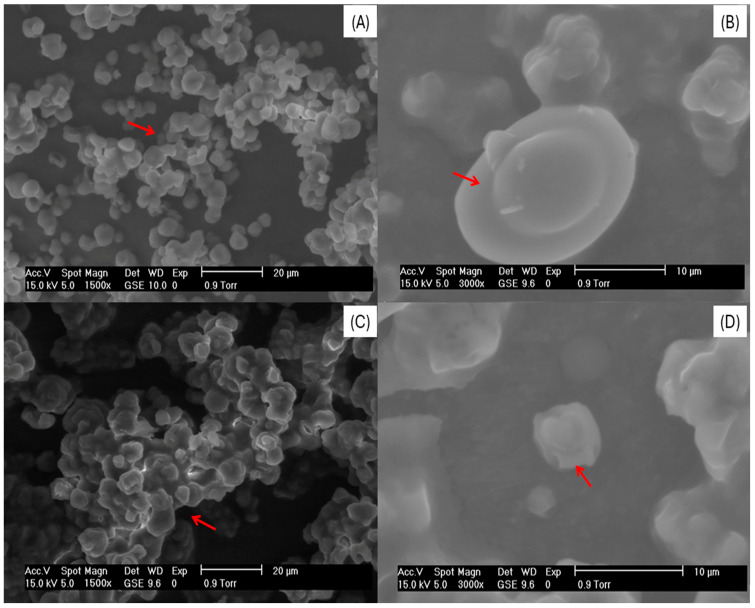
Non-plasmolyzed microcapsules at 1500× (**A**) and 3000× (**B**) and plasmolyzed microcapsules at 1500× (**C**) and 3000× (**D**). The red arrows in the figure indicate the presence of changes in the yeast cells.

Figure 1 shows the morphology of the non-plasmolyzed cell (NPC), plasmolyzed cell (PC), non-plasmolyzed microcapsule (NPM), and plasmolyzed microcapsule (PM). The NPC is shown in Figure 1A,B. The morphology is oval with rounded ends and regular surfaces, and the formation of the characteristic reproduction budding yeast cells was observed, which indicates that the cells are viable [40]. The PC (Figure 1C,D) displays agglomeration, deformation of the cell wall, and a decrease in size, as well as cells without signs of budding. The morphological differences between the NPC and PC are attributed to the plasmolysis process, which means the effect of the hypertonic NaCl solution causes the loss of water through osmosis, decreasing the size of the cell and the plasma membrane separating it from the cell wall, causing loss of turgor, forming concave areas in the cell wall, deforming the cell, and changing physical properties [41,42]. Morphological differences between the NPC and PC have already been reported in other studies, such as [43], where the *S. cerevisiae* yeast cells were lysed by different methods for comparative study. Plasmolysis was performed with 15% ethyl acetate, obtaining a deformation of the cell wall of the cells compared to live cells that were round, smooth, and turgid. Refs. [21,25,27] plasmolyzed yeast cells using a hypertonic 10% NaCl solution, where deformations and a significant decrease in cell size were also found. The NPM (Figure 2A,B) shows ovals and soft cells with slight swelling, agglomeration, and deformation in the cell wall caused by the spray drying process observed in comparison with the NPC (Figure 1A,B). The PM (Figure 2C,D) was observed as being more turgid, with more evident agglomeration and greater deformation in the structure of the cell wall. These results are attributed to the microencapsulation process; that is, the swelling is because the cells are hydrated in an aqueous solution with the extract, which can increase the turgor in the cells since, during drying, the water content is not eliminated inside the cell, and high temperatures of spray drying can significantly affect the structure of the cells by deforming them [44,45]. These results have already been reported by other authors using spray drying to microencapsulate bioactive compounds in *S. cerevisiae* yeast cells, plasmolyzed and non-plasmolyzed, such as in [25], which evaluated different solvent mixtures (ethanol:water) for the introduction of gallic acid into yeast cells and observed swelling in plasmolyzed cells and deformation in non-plasmolyzed cells after the microencapsulation process. Studies have already shown that NaCl plasmolysis provides more space for core loading by releasing water, some water-soluble ingredients such as proteins and nucleic acids, and some enzymes from the cell, thereby enhancing the encapsulation of compounds. Furthermore, other researchers also reported similar data on hydrophobic and hydrophilic substances loaded into *S. cerevisiae* yeast cells, increasing the intracellular space due to plasmolysis, resulting in the simultaneity of core loading with the release of cellular materials [10]. The drying process yield may vary depending on factors such as the properties of the material to be dried, operating parameters, and equipment design and maintenance.

### 3.3. Fourier Transform Infrared Spectroscopy (FTIR)

**Figure 3:**
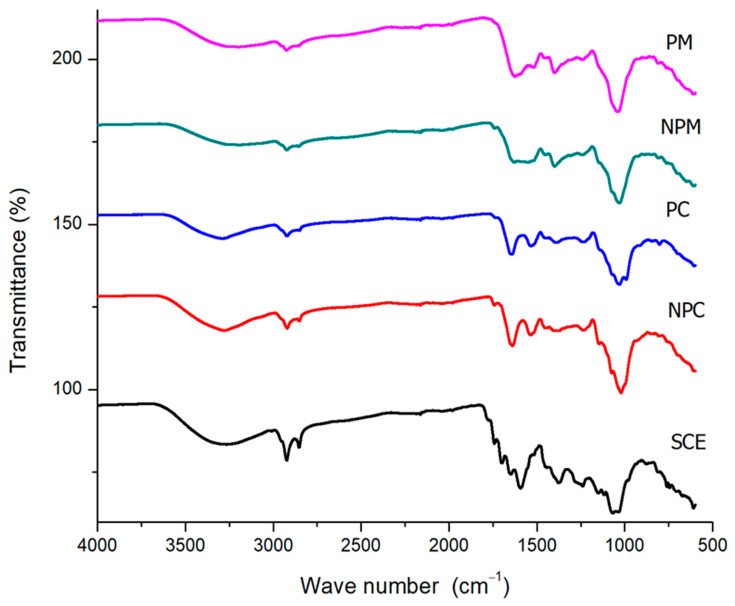
FTIR spectrum of spent coffee extract (SCE), non-plasmolyzed *Saccharomyces cerevisiae yeast* cells (NPCs), plasmolyzed *Saccharomyces cerevisiae* yeast cells (PCs), non-plasmolyzed microcapsules (NPMs), and plasmolyzed microcapsules (PMs).

To confirm the encapsulation of bioactive compounds into *Saccharomyces cerevisiae* yeast cells, FTIR was used. The FTIR spectrum of spent coffee extract (SCE), NPCs, PCs, NPMs, and PMs is shown in Figure 3. In SCE, a peak was observed at 3273 cm^−^^1^, attributed to the vibration of the O-H bond of the hydroxyl groups at 2924 and 2849 cm^−^^1^, suggesting the presence of C-H stretching vibrations of the CH_3_ and CH_2_ groups. At lower frequencies, a peak was observed at 1749 cm^−^^1^, characteristic of the stretching of the C-O double bond of the carboxyl group of ester compounds, and at 1649 cm^−^^1^, characteristic of the stretching of the C-C double bond of the aromatic ring of the phenol group. At 1595 cm^−^^1^, stretching bands of the C-C double bond appear, mainly attributed to caffeine. Finally, peaks were observed at 1238 cm^−^^1^ and 1032 cm^−^^1^, attributed to the stretching of the C-O ester bond and the characteristic sign of the presence of chlorogenic acid, respectively. These peaks in the SCE spectrum confirm the presence of the hydroxycinnamic acids identified in this investigation by HPLC, which are characterized by the phenol group and the carboxylic acid of the side chain, in addition to caffeine and chlorogenic acid (ester) [46]. These characteristic bands have already been observed in other studies, such as in [47], which evaluated spent coffee with drying pretreatment at 60 °C before the extraction of phenolic compounds. The authors reported the stretching of O-H at 3200 cm^−^^1^; at 1649 cm^−^^1^, the stretching of the C-C double bond of the aromatic groups of the phenol group is observed; and at 1026 cm^−^^1^, the confirmation of the presence of chlorogenic acid is observed. On the other hand, ref. [48] evaluated green coffee beans, obtaining peaks at 1742 cm^−^^1^ and 1115 cm^−^^1^ of the vibration corresponding to the ester bonds; however, compared to this study, a shift of the peaks is observed that may be due to the thermal and extraction process for obtaining spent coffee. The NPC’s bands were obtained at 3276 cm^−^^1^ of the O-H vibration of the hydroxyl group of the cell wall polysaccharides, at 2913 cm^−^^1^ and 2841 cm^−^^1^, indicating the vibration of the C-H stretching of the aliphatic chains of fatty acids, 1640 cm^−^^1^ and 1538 cm^−^^1^ characteristic of amide I and amide II of proteins, as well as the band at 1019 cm^−^^1^ characteristic of β-glucans. Results on the functional groups of yeast cell components have been reported: the authors of [27] plasmolyzed *S. cerevisiae* yeast cells with a 10% hypertonic NaCl solution and reported a shift from 3345 cm^−^^1^ to 3333 cm^−^^1^ in the vibration of the O-H bond of the hydroxyl group of polysaccharides after the plasmolysis process, and likewise, ref. [44] plasmolyzed *S. cerevisiae* yeast cells with 5% ethyl acetate and reported a shift in the hydroxyl group signal from 3200 cm^−^^1^ to 3250 cm^−^^1^. Regarding the PC, they show a slight shift in the peaks, which is attributed to the effect that plasmolysis has on the components of the cell wall, made up mainly of polysaccharides, mannoproteins, and simple fatty acids [49]. The NPM showed bands at 3274 cm^−^^1^ of the O-H vibration, 2924 and 2849 cm^−^^1^ of the C-H stretching vibration of fatty acids, and 1555 cm^−1^ of the amide I of proteins. The peak observed at 1031 cm^−^^1^ is characteristic of β-glucan. In comparison with the NPC, a decrease in the intensity of the peaks and the absence of the amide II peak in the NPM is observed, which is due to the possible interactions of the phenolic compounds with the components of the cell wall [50]. The interactions could be confirmed with the signal obtained from the β-glucans, which is more defined in the NPM than in the NPC due to the interference of the characteristic signal of chlorogenic acid. This could indicate a favorable interaction of the polysaccharides of the cell wall with the chlorogenic acids of the spent coffee extract. In the PM compared with the NPM, only a decrease in intensity was shown in the bands found but not a shift, which could indicate a greater interaction of the bioactive compounds with the cell wall compounds in the NPC. Compared to the PC, this may indicate that plasmolysis increased internal encapsulation and, thus, the stabilization of bioactive compounds [9].

### 3.4. Differential Scanning Calorimetry

**Figure 4:**
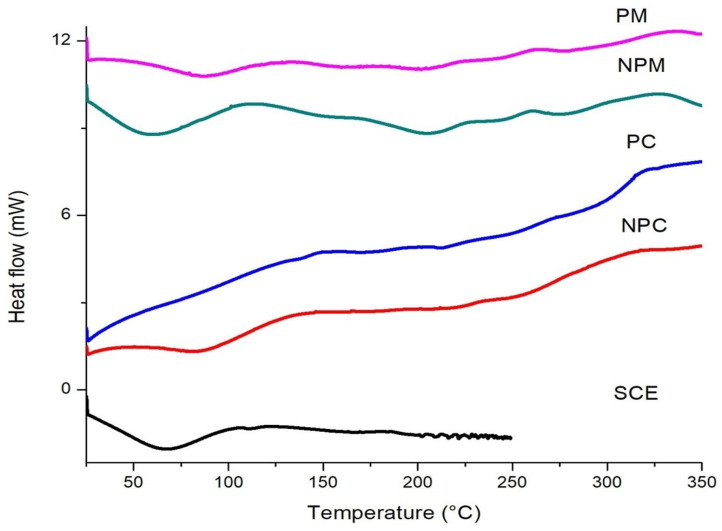
DSC thermogram of spent coffee extract (SCE), non-plasmolyzed *Saccharomyces cerevisiae* yeast cells (NPCs), plasmolyzed *Saccharomyces cerevisiae* yeast cells (PCs), non-plasmolyzed microcapsules (NPMs) and plasmolyzed microcapsules (PMs).

The thermal analysis of the SCE, NPC, PC, NPM, and PM is shown in Figure 4. In the SCE, an endothermic phase event was obtained at 67.5 °C, related to the vaporization of water, and another at 105 °C of the total degradation of the phenolic compounds [15,51].

Ulloa et al. [52] used a method with alkali and autohydrolysis to extract the bioactive compounds from coffee bagasse and obtained a thermogram with an endothermic peak at 76.89 °C and 100.90 °C with degradation that begins at 118 °C and 198 °C, respectively. The differences in the thermal degradation of the bioactive compounds with the present investigation are mainly due to the extraction method. The NPC showed two endothermic peaks, one broad at 86 °C corresponding to the temperature of water vaporization and another less intense one at 174.9 °C corresponding to the fusion of the phospholipid bilayer, also showing a total cellular degradation at 320.21 °C. The PC, compared to the NPC, does not show the endothermic peak of water vaporization but does show a shift in the fusion of phospholipids, decreasing the temperature to 135 °C. This is due to the plasmolysis process, which causes cellular dehydration and deformation of the plasma membrane, so phospholipids can have a much lower phase transition temperature from gel to liquid crystalline [52,53]. Studies such as [27] report temperatures of 62 °C in the vaporization event and 200 °C for the fusion of phospholipids, and [54] reported a melting temperature of 207.88 °C for active *S. cerevisiae* yeast cells. Likewise, the PM, compared to the PC, shows an endothermic peak at 87 °C related to the vaporization of water from the PM, which can be attributed to the rehydration of the cell during the microencapsulation process. Regarding the NPM and PM, at 147 °C, this can be attributed to the degradation of the bioactive compounds of the spent coffee extract and, finally, at 205 °C from the fusion of the phospholipids of the plasma membrane. Furthermore, the degradation of antioxidants from the SCE compared to that of the NPM and PM would indicate that there is an increase in the degradation temperature of bioactive compounds observed in the microcapsules, so the yeast cell is stabilizing the bioactive compounds with relation to the increase in temperature, which confirms the FTIR results of the interactions of the spent coffee extract with the structure of the *S. cerevisiae* yeast cells.

### 3.5. Encapsulation Efficiency (EE) and Load Capacity (LC)

**Figure 5:**
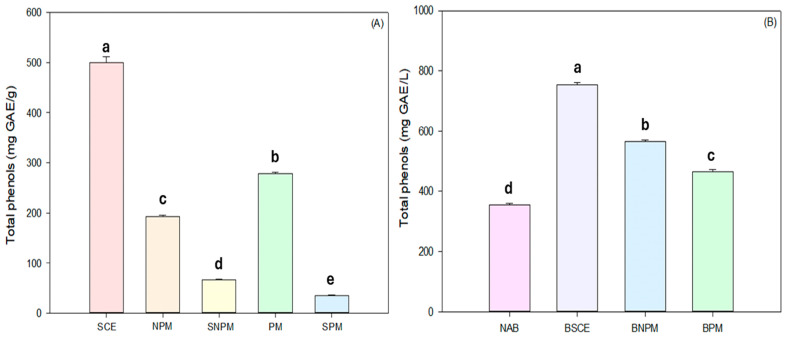
(**A**) Quantification of total phenols in spent coffee extract (SCE), non-plasmolyzed microcapsules (NPMs), plasmolyzed microcapsules (PMs), superficial non-plasmolyzed microcapsules (SNPMs), and superficial plasmolyzed microcapsules (SPMs). (**B**) Non-additive beer (NAB), beer with spent coffee extract (BSCE), beer with non-plasmolyzed microcapsules (BNPM), and beer with plasmolyzed microcapsules (BPM). Mean ± standard deviation. Different letters (a,b,c,d,e) between treatments are significantly different if *p* < 0.05.

The results obtained from the total phenol quantification of the SCE, PM, NPM, superficial non-plasmolyzed microcapsule (SNPM), and superficial plasmolyzed microcapsule (SPM) are shown in Figure 5A. A higher concentration of total phenols was observed in the SNPM (66.45 mg EAG/g dw) compared to the SPM (35.88 mg EAG/g dw); however, there was a higher concentration in the PM (278.47 mg EAG/g dw) compared to the NPM (192.8 mg EAG/g dw) after the sonication process. The results can be attributed to plasmolysis treatment in the cells since there is a greater intracellular space and, above all, there is greater permeability of the plasma membrane, which allows the passage of caffeine and phenolic acids present in the spent coffee extract [27,45]. The effect of plasmolysis can also be confirmed on the loading capacity of bioactive compounds with further quantification of the encapsulation efficiency (EE) and loading capacity (LC) [49,51]. Table 2 shows the total phenolic content inside the cell and the encapsulation efficiency. Several authors have already reported results using plasmolyzed yeast cells, such as the study reported by [27], which microencapsulated purslane seed oil in non-plasmolyzed *Saccharomyces cerevisiae* yeast cells and plasmolyzed cells, reporting an encapsulation efficiency of 52.96–60.27% and a loading capacity of 186.87–211.68 g/Kg, respectively. On the other hand, ref. [51] reported an encapsulation efficiency of 23.10–43.10% and a loading capacity of 111.02–216.52 g/Kg. In comparison, both investigations have similarities that are attributed to the plasmolysis treatment benefiting the intracellular interaction and increasing the encapsulation yield.

### 3.6. Digestion of Microcapsules Added to a Craft Beer

The quantification of total phenolics of non-additive beer (NAB), beer with spent coffee extracts (BSCE), beer with non-plasmolyzed microcapsules (BNPM), and beer with plasmolyzed microcapsules (BPM) is shown in Figure 5B. The NAB has a lower concentration of total phenols (353.68 ± 3.88 mg EAG/L), which, compared to the BSCE, shows a significant increase (*p* < 0.05) in the concentration of total phenols in the beer up to 747.45 ± 3.36 mg EAG/L after the addition of unencapsulated spent coffee extract. In the beer samples with microcapsules, a greater increase in the quantification of phenols was observed in the BNPM (564.87 ± 3.45 mg EAG/L) compared to the BPM (463.94 ± 7.36 mg EAG/L), as already previously demonstrated. The NPM has a higher concentration of total phenols on the cell surface, which allows greater interaction with the environment and greater total phenol quantification before digestion. The addition of extracts in craft beers has already been reported to increase the antioxidant properties of the drink, as shown in [52], which added propolis extract in a craft beer in different concentrations to maximize the addition of phenolic compounds, obtaining an increase in total phenols from 242 to 306.5 mg EAG/L. Compared to this study, a smaller increase in the concentration of total phenols is observed after the addition of the unencapsulated extract, which is due to the nature of the raw material used to extract the bioactive compounds. The effects of the stomach, intestinal, and dialysis phases on the bioaccessibility of total phenols in the NAB, BSCE, BNPM, and BPM are shown in Table 3. The NAB and BSCE showed a significant decrease (*p* < 0.05) as the digestive phases progressed, obtaining an intestinal bioaccessibility of 38.78 and 39.65%, respectively. This is attributable to the interactions of phenolic compounds with their environment, which includes ionic and covalent interactions with other molecules present from the food matrix or the digestive phase, enzymatic actions of lipase and pepsin, and the change in pH, which causes the degradation of bioactive compounds in spent coffee [53]. The effect of the digestive phases on the bioaccessibility of bioactive compounds has already been evaluated by other authors in different foods and beverages, such as in [54], which evaluated the effect of digestion on the bioaccessibility of phenolic compounds in grape pomace, obtaining a bioaccessibility of 122% in the intestinal phase; as did [55], which obtained similar results with organic pumpkin pomace with 116.3% bioaccessibility in the intestinal phase. Compared with this study, a much lower bioaccessibility was obtained in the intestinal phase in the NAB and BSCE samples. A similar trend is observed by [56] in the evaluation of the bioaccessibility of red wine through digestion, which reported a decrease as the digestion process progressed, with the loss of phenols being more noticeable in the gastric phase. Likewise, ref. [57] evaluated the bioaccessibility of coffee, observing a decrease in the concentration of phenols throughout the digestive stages, similar to this research. Bioaccessibility values greater than 100% indicate that bioactive compounds were released from the food matrix and/or metabolized from more complex bioactive compounds. Enzymes, pH, and the action of bile salts in the stomach and intestinal environment can cause variations in the chemical structures of phenolic compounds, giving rise to new molecules with different bioavailability and biological activities; therefore, the differences and similarities are due to the concentration of free and conjugated phenols in the food matrix [58]. Solid foods are mainly composed of conjugated phenols that can be released by enzymatic action, while beverages are rich in free phenolic compounds, which allow greater exposure to physical and chemical interactions with their environment and can cause the degradation of bioactive compounds [59]. In the BNPM and BPM samples, significant differences (*p* < 0.05) were observed in the digestive stages; however, unlike the NAB and BSCE, the beers with microcapsules showed increased bioaccessibility of bioactive compounds in the intestinal phase: 96.79% in the BNPM and 107.81% in the BPM. It can be observed that during the gastric phase, there is a decrease in phenolic compounds that may be due to physical and chemical interactions with the environment. This is where enzymes (pepsin and lipase) begin to degrade components of the yeast cell wall, such as fatty acids. Upon reaching the intestinal phase, the *Saccharomyces cerevisiae* yeast cell structure collapses when it interacts with the complex of pancreatin used during in vitro digestion. Pancreatin, which is secreted by the pancreas in a living system, hydrolyzes proteins and lipids by emulating the membrane of cellular phospholipids that already have structural damage due to plasmolysis, facilitating the enzymatic breakdown of the cellular structure [8]. In all samples, a bioaccessibility above 90% was observed after dialysis, indicating that the total phenols found in the intestinal phase have a high percentage of being absorbed by passive diffusion through the intestine [60]. The results obtained can be related to the stability of the bioactive compounds of the spent coffee extract microencapsulated in yeast cells against environmental factors that can degrade them. This shows better encapsulation yields and greater bioaccessibility of the bioactive compounds in the PMs because they have a higher fraction at the intracellular level compared to non-plasmolyzed cells.

The changes in the quantification of antioxidant activity subject to the digestive phases in the samples of the NAB, BSCE, BNPM, and BPM are shown in Table 3. A decrease in the antioxidant activity was observed as the digestive phases progressed in the NAB and BSCE samples; conversely, in the samples of BNPM and BPM, there was an increase in antioxidant activity in the intestinal phase. In addition, it can be seen that there is a high correlation between the total phenols and the quantification of the antioxidant activity evaluated by means of a Pearson correlation with *p* < 0.05, slightly larger with ABTS^•+^ with a value of R = 0.91, followed by DPPH with R = 0.89 and FRAP with R = 0.79. This indicates that spent coffee extract compounds, such as chlorogenic acid and caffeine, can provide most of the antioxidant activity with hydrogen atom transfer (HAT) mechanisms and hydrophilic compounds in comparison with DPPH^•^, which mostly responds to hydrophobic compounds, and FRAP, with a mechanism of single electron transfer mechanism (SET) actions. However, Pearson’s correlation is not completely linear, so the antioxidant activity, although mainly provided by the bioactive compounds of the microencapsulated coffee extract, is also provided to some degree by the bioactive components of the beer. Also, the results of the microencapsulation digestion of the bioactive compounds of spent coffee indicate that the compounds are being stabilized by yeast cells and released during the intestinal phase, where a greater absorption of phenolic compounds through the barrier (microvillus) of the intestine can occur by several mechanisms, including passive diffusion [16,52,60].

## 4. Conclusions

The *Saccharomyces cerevisiae* yeast cells functioned as an encapsulating wall material, stabilizing the bioactive compounds of the spent coffee extract against environmental factors such as high temperatures. In addition, plasmolysis increased the efficiency of encapsulation and the loading capacity in microcapsules. Microencapsulated *Saccharomyces cerevisiae* yeast cells managed to protect and direct the bioactive compounds of spent coffee extract added to a craft beer during in vitro digestion. The addition of the spent coffee extract and microcapsules increased the functionality of the beer. These microcapsules could be used in dark beers and other foods or beverages that require an increase in their functional properties. Therefore, the microcapsules obtained could be used as a functional additive in the food industry.

## Figures and Tables

**Table 1:** Identification and quantification of phenolic compounds and caffeine from spent coffee extract.

Bioactive Compound	λ Max	tR	[µg/g]	LOD [µg/g]	LOQ [µg/g]	R^2^
Gallic acid	270	3.53	448.02 ± 5.74	284	661	0.999
Chlorogenic acid	330	18.46	4349.42 ± 4.66	850	2577	0.993
Cafeic acid	320	20.35	880.14 ± 4.07	285	738	0.997
Caffeine	270	20.12	28,919.13 ± 4.18	4242	12,859	0.996
Siringic acid	320	22.09	412.87 ± 2.89	117	386	0.998
p-cumaric acid	320	26.15	583.91 ± 6.34	186	370	0.975
Ferulic acid	320	30.15	484.23 ± 6.83	114	347	0.976
Sinapinic acid	320	31.71	787.19 ± 4.66	122	565	0.982

Mean ± standard deviation, *n* = 3. LOD: Limit of Detection; LOQ: Limit of Quantification. The LOD and LOQ were based on the linear regression coefficient of the calibration curves of each standard with the equations LOD = 3.3 σ/S and LOQ = 10 σ/S.

**Table 2:** Encapsulation efficiency and loading capacity in microcapsules.

Sample	EE (%)	LC (g/Kg)
NPM	38.32 ^b^ ± 0.32	246.96 ^b^ ± 8.41
PM	56.56 ^a^ ± 0.64	490.34 ^a^ ± 8.41

Mean ± standard deviation. Different letters (a,b) between treatments are significantly different if *p* < 0.05. Non-plasmolyzed microcapsules (NPMs) and plasmolyzed microcapsules (PMs).

**Table 3:** Quantification of total phenols, bioaccessibility, and antioxidant activity of craft beers after in vitro digestion.

Phase	Sample	Total Phenols (mg GAE/L)	Bioaccessibility (%)	DPPH^•^ (mMol TE/L)	ABTS^•+^ (mMol TE/L)	FRAP (mMol TE/L)
Gastric	NAB	213.55 ^b^ ± 1.56	60.37	0.78 ^b^ ± 0.02	0.58 ^b^ ± 0.10	0.25 ^b^ ± 0.03
	BSCE	459.56 ^b^ ± 2.13	61.48	1.32 ^b^ ± 0.24	0.96 ^b^ ± 0.27	0.58 ^b^ ± 0.06
	BNPM	365.11 ^b^ ± 2.67	64.67	1.09 ^c^ ± 0.13	0.86 ^c^ ± 0.17	0.9 ^c^ ± 0.08
	BPM	302.13 ^c^ ± 2.87	65.17	0.89 ^c^ ± 0.04	0.74 ^c^ ± 0.14	0.8 ^c^ ± 0.05
Intestinal	NAB	137.16 ^c^ ± 2.24	38.78	0.31 ^c^ ± 0.01	0.24 ^c^ ± 0.03	0.09 ^c^ ± 0.01
	BSCE	296.37 ^c^ ± 1.38	39.65	0.71 ^c^ ± 0.09	0.34 ^c^ ± 0.09	0.14 ^c^ ± 0.02
	BNPM	546.43 ^a^ ± 2.6	96.79	1.44 ^a^ ± 0.07	0.87 ^a^ ± 0.06	0.89 ^a^ ± 0.13
	BPM	499.74 ^a^ ± 1.43	107.81	1.39 ^a^ ± 0.32	1.01 ^a^ ± 0.17	1.09 ^a^ ± 0.26
Dialysis	NAB	30.29 ^d^ ± 1.72	91.43	0.09 ^d^ ± 0.01	0.02 ^d^ ± 0.01	0.01 ^d^ ± 0.00
	BSCE	53.19 ^d^ ± 1.93	92.88	0.14 ^d^ ± 0.03	0.06 ^d^ ± 0.01	0.05 ^d^ ± 0.01
	BNPM	46.6 ^d^ ± 0.87	91.74	0. 09 ^d^ ± 0.01	0.02 ^d^ ± 0.01	0. 02 ^d^ ± 0.01
	BPM	35.34 ^d^ ± 0.33	92.37	0.11 ^d^ ± 0.01	0.04 ^d^ ± 0.01	0.03 ^d^ ± 0.01

Mean ± standard deviation. Different letters (a,b,c,d) between treatments are significantly different if *p* < 0.05. Non-additive beer (NAB), beer with spent coffee extract (BSCE), beer with non-plasmolyzed microcapsules (BNPM), and beer with plasmolyzed microcapsules (BPM).

## Data Availability

The data presented in this study are available on request from the corresponding author because the data are part of an ongoing study, and the results have not yet been published. The raw data supporting the conclusions of this article will be made available by the authors on request.

## Abbreviations

The following abbreviations are used in this manuscript:

NPC	Non-plasmolyzed cell
PC	Plasmolyzed cell
NPM	Non-plasmolyzed microcapsule
PM	Plasmolyzed microcapsule
NAB	Non-additive beer
BSCE	Beer with spent coffee extract
BNPM	Beer with non-plasmolyzed microcapsules
BPM	Beer with plasmolyzed microcapsules
EE	Encapsulation efficiency
LC	Load capacity
SCE	spent coffee extract
SPM	superficial plasmolyzed microcapsule
SNPM	superficial non-plasmolyzed microcapsule
